# Quantitative Analysis of Chiari-Like Malformation and Syringomyelia in the Griffon Bruxellois Dog

**DOI:** 10.1371/journal.pone.0088120

**Published:** 2014-02-12

**Authors:** Susan P. Knowler, Angus K. McFadyen, Courtenay Freeman, Marc Kent, Simon R. Platt, Zoha Kibar, Clare Rusbridge

**Affiliations:** 1 Neurology Department, Fitzpatrick Referrals, Godalming, Surrey, United Kingdom; 2 akm-stats, Glasgow, Scotland, United Kingdom; 3 Department of Small Animal Medicine & Surgery, College of Veterinary Medicine, University of Georgia, Athens, Georgia, United States of America; 4 Department of Neurosciences, Justine University Hospital Research Center, University of Montreal, Quebec, Canada; 5 School of Veterinary Medicine, Faculty of Health & Medical Sciences, University of Surrey, Guildford, United Kingdom; University of Sydney, Australia

## Abstract

This study aimed to develop a system of quantitative analysis of canine Chiari-like malformation and syringomyelia on variable quality MRI. We made a series of measurements from magnetic resonance DICOM images from Griffon Bruxellois dogs with and without Chiari-like malformation and syringomyelia and identified several significant variables. We found that in the Griffon Bruxellois dog, Chiari-like malformation is characterized by an apparent shortening of the entire cranial base and possibly by increased proximity of the atlas to the occiput. As a compensatory change, there appears to be an increased height of the rostral cranial cavity with lengthening of the dorsal cranial vault and considerable reorganization of the brain parenchyma including ventral deviation of the olfactory bulbs and rostral invagination of the cerebellum under the occipital lobes.

## Introduction

Canine Chiari-like malformation (CM) is a complex abnormality characterized by overcrowding of the craniocervical junction and by disparity in size between the brain parenchyma (too big) and the skull (too small) [1]. CM is common in several brachycephalic toy breeds including Cavalier King Charles spaniel and crosses, King Charles spaniel, Griffon Bruxellois, Affenpinschers and Chihuahuas [1]. It has been proposed that CM is due to occipital bone insufficiency and an inadequate caudal cranial fossa volume [2]–[5]. Moreover, Cavalier King Charles spaniels have relatively increased cerebellar volume compared to other breeds and overcrowding of the cerebellum in the caudal part of the caudal cranial fossa is correlated with the development of syringomyelia [6]–[9]. CM and the consequent neural parenchymal overcrowding with obstruction of the cerebrospinal fluid channels predisposes affected dogs to the painful spinal cord disease syringomyelia [10]. Syringomyelia has a variable age of onset and dogs with a more severe phenotype develop syringomyelia as young dogs [2]. By 3 years of age the majority of dogs predisposed to syringomyelia will have MRI evidence of it and only a small percentage of predisposed dogs that are MRI clear of syringomyelia at 3 years of age develop MRI signs later. In these older dogs the MRI changes are typically mild for example a central canal dilatation [11].

CM is assumed to be a developmental disorder. The rostral portion of the cranial base is of neural crest origin whereas the caudal portion is of mesodermal origin [12], [13]. The boundary between these regions ultimately becomes the junction of the basisphenoid and basioccipital bones [12]. The cranial base is the first part of the skull to develop and begins as mesenchyme condensations beneath the brain and around the developing sensory organs [12]. This forms a longitudinal cartilage trough which remodels and adapts to the developing neural structures [12], [14]. Expansion of the cranial base occurs by primary growth of the cartilage and by expansion at the synchondroses i.e. at the suture lines. Growth is uneven, reflecting and accommodating the developing brain. The rostral and caudal sections (divided by the hypophyseal fossa or sella turcica) increase their length at different rates and at different times with the sphenoid and basioccipital bones developing more slowly. The spheno-occipital synchondrosis makes a significant contribution to growth post-natally [12]. A recent study found that the spheno-occipital synchondrosis was closed in 80% of dogs at 4 months old and that this suture seemed to ossify earlier in Cavalier King Charles spaniels compared to other brachycephalic dogs, which in turn ossified earlier than mesaticephalic dogs [15]. The cranial base is angled at the level of the hypophyseal fossa where the rostral prechordal and caudal chordal parts meet. In the early embryo this angle is very obtuse but by the time of ossification it should have flattened. Consequently inadequacy of cartilage growth will result in a short cranial base with increased angulation [16]. In humans, the clivus (basioccipital)–supraocciput angle is a useful parameter to differentiate various causes of fetal ventriculomegaly and in particular to recognize Chiari type II malformation [17]. However, it has not been established whether brachycephalic toy breed dogs with CM are more likely to have a shortened and/or angled cranial base compared to other toy breed dogs. At least five regions of the canine genome are associated with brachycephaly [18]. A missense mutation in bone morphogenetic protein 3 (*BMP3*) that is nearly fixed among extreme brachycephalic toy dog breeds including the Griffon Bruxellois and the Cavalier King Charles spaniel has been identified as a major contributor [18], [19]. *BMP3* is highly expressed at the synchondroses, suggesting a role in chondrogenesis [18]–[20].

CM is a complex disorder and although there is less phenotypic variation than with humans, there can be differences between breeds and individuals within the same breed. In particular the conformation of the craniocervical junction varies and in some individuals the size of cerebellar herniation may be minimal [3]. This feature in particular makes CM dissimilar from the analogous human condition Chiari type I malformation although there are some similarities to Chiari type 0 malformation [21]. The term craniovertebral junction refers to the bony structures surrounding the medulla oblongata, the cervicomedullary junction and the upper cervical spinal cord and is constructed of the occipital bones forming the foramen magnum, the atlas and the axis. Mechanically the craniocervical junction consists of a central pivot (basioccipital bone, dens and axis) and two rings (foramen magnum and atlas). The embryology mirrors the functionality with the central pillar originating from the axial portion of the occipital and first two cervical sclerotomes, whereas the ring structures come from the lateral portion of the first two cervical sclerotomes [22]. As a consequence disturbances in development of the axial portion may result in anomalies of the dens pivot and the basiocciput and disturbances in the development of the lateral portion may result in abnormalities of the occipital condyles, atlas arch and lateral masses of the atlas and axis [22]. In other words a developmental anomaly resulting in a Chiari malformation may also be associated with abnormalities of the atlas, axis and dens. In the dog, the most important craniovertebral junction abnormality associated with CM is atlanto-occipital overlapping, which has been reported as similar to basilar invagination in humans [23], [24]. Both conditions are characterized by increased proximity of the cranial cervical spine to the base of the skull [25]; however a defining characteristic of human basilar invagination is invagination of the odontoid process of the axis through or towards the foramen magnum, often with compression of the neural tissue by the dens [25]. Other less common canine craniovertebral junction anomalies include atlantoaxial subluxation [26], [27] and dorsal angulation of the dens [28]. Occipital dysplasia (i.e. widened foramen magnum) also may be seen [29]; however this is probably an acquired condition due to overcrowding of the caudal cranial fossa, mechanical pressure from the cerebellum and supraoccipital bone resorption [30].

These variations in morphology, and the undetermined significance of their contribution to CM, compound the difficulty in diagnosing the condition and accurate assessment of the risk of a dog developing syringomyelia and/or passing on this risk to offspring. It also creates difficulties in accurate phenotyping for genetic studies.

The Griffon Bruxellois (known as the Brussels Griffon in the USA) has a high prevalence of the disorder with a conservative estimate of 65% having CM and 42–52% having syringomyelia [3], [31]. Accurate phenotyping is pivotal to facilitate genetic studies into this disorder.

The diagnosis of CM and syringomyelia is dependent on magnetic resonance imaging (MRI) of the brain and cervical spinal cord. In ideal circumstances, an experienced operator would image all the subjects in a high field MRI unit obtaining excellent quality images enabling a quantitative assessment of the morphology. In reality the cohort for this group’s genetic studies is sourced from a global population of dogs undergoing imaging for diagnostic and screening prior to breeding purposes. Although the dogs are imaged with the same basic protocol, the tesla strength of the MRI unit and the skill of the operators have varied considerably. Another difficulty is that for economic reasons, assessment of breeding dogs is made from only 3 MRI sequences of a limited anatomical area that does not include the entire cranial fossa [32]. The aim of this study was to develop a consistent system of measurements that could provide a quantitative analysis of the CM and syringomyelia phenotype from a single sagittal MRI sequence obtained by machines of differing field strength to enable further genetic studies and facilitate improved understanding of the pathogenesis of the condition. We achieved this aim and found several significant variables that have enabled the genetic study to progress.

### Ethics Statement

This study did not involve live animals but the analysis of MRI DICOM obtained for diagnostic purposes or for determining CM and syringomyelia status. Full consent was obtained from all owners and actual dogs remained anonymous.

## Materials and Methods

**Figure 1:**
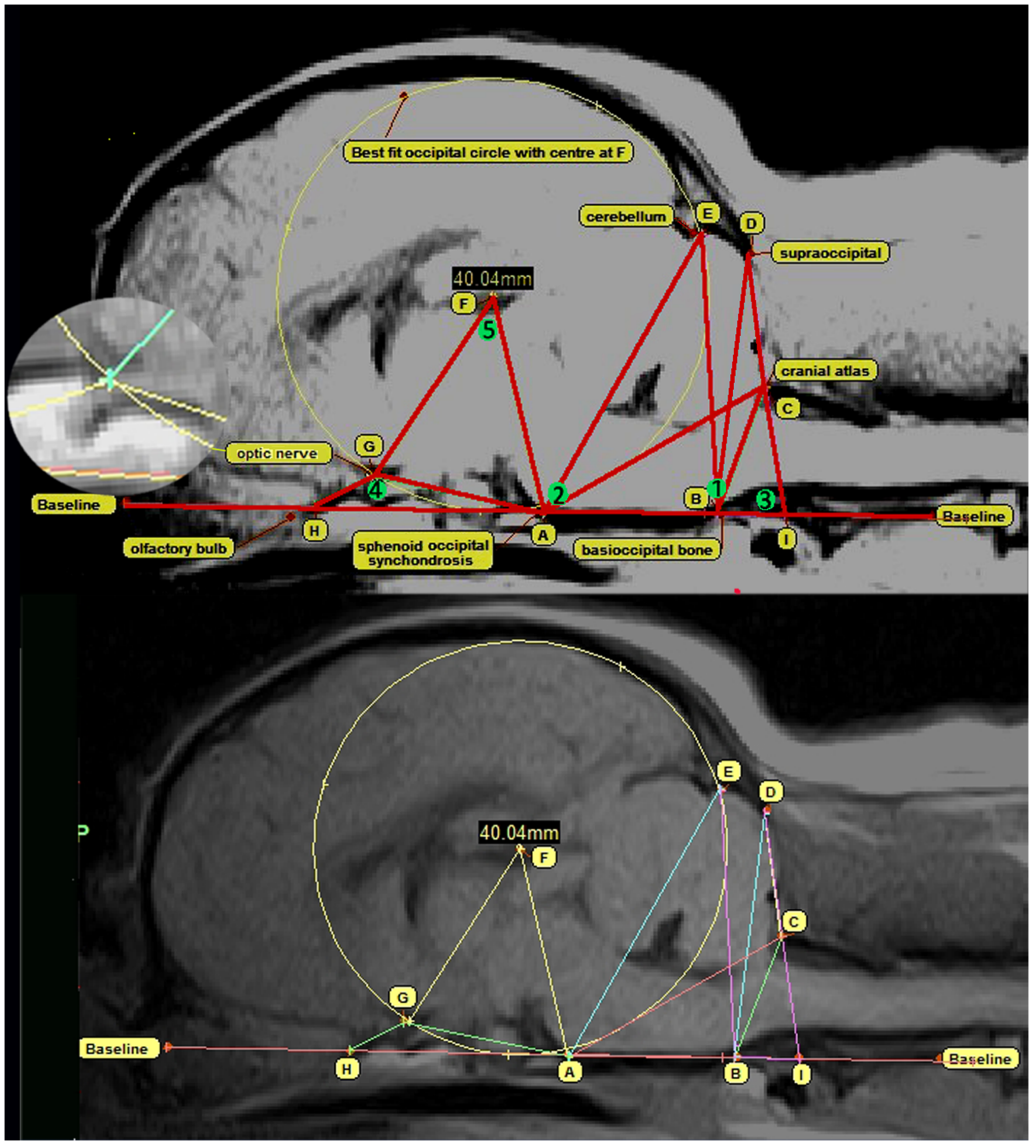
Midline sagittal T1-weighted MRI of hindbrain and craniocervical region of a three year old female Griffon Bruxellois with the framework of measured lines and angles assessing conformational features associated with CM. The upper image has been “windowed” to create improved contrast and highlight bony landmarks with a magnification of the area of the optic nerve and canal (inset). The lower image demonstrates the same without the windowing effect. All line measurements start from one of 9 points: (A) dorsum of spheno-occipital synchondrosis. (B) basion of basioccipital bone. (C) rostral edge of the dorsal lamina of the atlas. (D) junction between the supraoccipital bone and the occipital crest. (E) most dorsal point of intersection of the cerebellum with the occipital lobe circle. (F) center of occipital lobe circle placed on the cranial baseline (HAI) and extending to encompass the occipital lobes. The centre of the circle is F. (G) point at which the optic nerve deviates into the canal (inset). (H) most caudal point of the olfactory bulb. (I) intersection point with the extended HA baseline. 5 angles measured are (1) ABC, (2) CAF, (3) AID, (4) AGH and (5) AFG.

The investigation reviewed 155 Digital Imaging and Communications in Medicine (DICOM) MRIs of the brain and cervical region of Griffon Bruxellois dogs. In addition, images from 6 other dogs were measured to provide a comparison for the study. This comprised of 3 mesaticephalic dogs (Beagle and 2 Australian terriers) and 3 Affenpinschers, a brachycephalic toy dog breed that is genetically close to the Griffon Bruxellois. The images had been obtained for diagnostic reasons (for example in the investigation for neurological signs and pain), for screening prior to breeding [31] or for screening to determine syringomyelia prevalence [33]. A minimal inclusion criterion was T1-weighted sagittal and transverse images of the brain and cranial cervical spinal cord although for many dogs the imaging protocol was more comprehensive. The strength of the MRI machines ranged from 0.2 to 3 Tesla. T1-weighted images were selected for the study, as these were the most diagnostically useful images from the low field MRI studies. The exception was images from a 3 Tesla system as the signal-to-noise ratio was suboptimal for standard T1-weighted images and so T1-weighted fluid attenuated inversion recovery (T1W FLAIR) images were used. The images were viewed using an eFILM DICOM viewer workstation 3.1 (Merge Healthcare https://estore.merge.com/na/index.aspx) by one of the authors (CR) who assigned a unique reference number to each dog and determined CM and syringomyelia status according to the British Veterinary Association/Kennel Club Chiari Malformation and Syringomyelia Health Scheme (Table 1) This grading system also takes into account the dog’s age at the age at time of MRI [32]. The DICOM images were then loaded into a software package MIMICS 14.12 Materialise (Technologielaan 15 3001 Leuven Belgium). This software has a windowing facility to control the image contrast hence enabling consistent identification of bony landmarks and, in addition, precise tools to measure the juxtaposition of the cerebrum and cerebellum and generate three-dimensional ‘masks’ of the brain. To investigate possible changes in the relative position of the atlas, spheno-occipital synchondrosis, cerebellum and optic canal, a framework comprising a circle and ten lines was constructed on midline sagittal T1-weighted images. Five angles, all associated with point A (dorsum of the spheno-occipital synchondrosis), within this framework were measured so that the brain and cranial cervical region could be ‘mapped’ in two dimensions (Figure 1). Earlier unpublished pilot studies had suggested that height of the rostral and caudal cranial fossa may be an important variable. However accurate assessment of this is hampered if the MRI does not include the rostral skull. To overcome this problem the authors used a circle extending from the cranial base line (HAI) and encompassing the caudal aspect of the occipital lobe. As this circle occupied the space between the base of the skull and the dorsum of the occipital lobe, the diameter (F-diameter) could be used as a reflection of the height of the rostral cranial cavity. The center of the circle (F) was used as a vertex and the occipital circle could also be used as an assessment of the size of the caudal cranial fossa by measuring the distance and angulation of the point where the circle bisected to the dorsal cerebellum (E). The latter overcame the problem of inconsistently identifiable caudal cranium bony landmarks which had been a problem in previous studies [34].

**Table 1:** Grading of Chiari-like malformation and syringomyelia according to the British Veterinary Association/Kennel Club Chiari-like malformation and Syringomyelia Health Scheme.

Chiari-like malformation (CM)	
Grade	
**0**	No Chiari-like malformation
**1**	Cerebellum indented (not rounded)
**2**	Cerebellum impacted into, or herniated through, the foramen magnum.
**Syringomyelia (SM)**	
**Grade**	
**0**	Normal (no central canal dilation, no presyrinx, no syrinx)
**1**	central canal dilation or a separate syrinx
	internal diameter of less than 2 mm or a pre-syrinx alone
**2**	**syringomyelia:**
	central canal dilation which has an internal diameter of 2 mm or greater
	a separate syrinx or pre-syrinx with central canal dilation
	(measured as the maximum internal diameter in a transverse plane)
The syringomyelia grade is qualified with a letter indicating the age group	
**Age group at time of scanning**	
**a**	more than five years of age
**b**	three to five years
**c**	one to three years of age.

**Syringomyelia** is defined as a fluid-filled cavity that includes or is distinct from the central canal of the spinal.

**Pre-syrinx** is defined as spinal cord oedema, and may be a transitional state prior to development of syringomyelia. Pre-syrinx has the appearance of high signal intensity on T2-weighted images consistent with marked increased fluid content within the spinal cord substance but not of free fluid. On T1-weighted images the spinal cord is either normal or has a slightly hypointense signal [32].

All MRI measurements were made by author SPK who was blinded to the CM and syringomyelia status and the identity of the dog. Reliability of the MRI measurements was assessed as intra-measurer reliability using intraclass correlation coefficient (ICC) Model (2, 1) [35]. Two measurements line AE and circle F, were recorded for ten dogs. Assuming reasonable reliability, a power of 80% was deemed achievable at the 5% level of significance if nine dogs were measured twice. To identify which MRI measurements would best help distinguish groups, multiple discriminant analysis models were developed. The first was for CM0 (No CM), CM1 (Mild CM) and CM2 (CM), the second for syringomyelia “Yes” and “No”. Syringomyelia may be a late onset condition and syringomyelia “No” dogs under 3 years of age were excluded from statistical calculations because syringomyelia clear status could not be assured. Conversely syringomyelia “yes” dogs under 3 years old were included as identifying conformational and genetic indicators of this severe phenotype is a paramount goal of the study.

The available data were used to develop discriminant functions for each analysis and then evaluated using a cross-validation technique thus avoiding data bias. Follow-up receiver operating characteristic (ROC) analysis on sensitivity, specificity, positive predictive value (PPV) and negative predictive value (NPV) was performed in an effort to assess clinical diagnostic relevance. The PPV and NPV in particular, allow the estimation of the proportions, respectively, of positives that are true positives and negatives that are true negatives. Microsoft Excel 2007 (http://office.microsoft.com/en-us/excel/) was used for data recording and manipulation and all statistical analysis was performed on SPSS v20 (http://www.ibm.com/software/analytics/spss) or Minitab v16 (http://www.minitab.com/en-us ) at the 5% level of significance i.e. p-values less than 0.05 were considered statistically significant.

## Results

### Study Dogs

There was very high intra-measurer reliability for the line AE data, with an ICC of 0.985, 95% CI (0.944, 0.996) and for the F-diameter data with an ICC of 0.880, 95% CI (0.591, 0.969). The 155 dogs in the study ranged from 1.1 to 12.6 years old (mean 4.5 years, standard deviation 2.5 years; median 3.8 years). 53 (34%) dogs were under three years of age. 39 (25%) were 3.0- to 4.9 years 63 (41%) were 5 years and over. Five dogs had insufficient imaging of the forebrain to identify the optic nerve and optic canal and, for 11 dogs, the quality of the scan prevented the olfactory bulbs from being identified with certainty so that line AH and Angle 5 were not recorded for these dogs.

The distribution of CM and syringomyelia affectedness is depicted in Table 2. Since syringomyelia may have a late onset, 20/53 dogs under three years with no central canal dilatation or syringomyelia were considered too young for a reliable syringomyelia status leaving a total of 135 dogs with confirmed syringomyelia status possible for this group.

**Table 2:** Number of dogs within degrees 0, 1, 2 for Chiari-like malformation (CM) and Syringomyelia (SM).

Number of dogs (%)			
CM		SM	
CM0	31 (20%)	SM0	35 (26%)
CM1	32 (21%)	SM1	33 (24%)
CM2	92 (59%)	SM2	67 (50%)
Total dogs	155		135

CM0 - no CM; CM1 - cerebellum indented by supraoccipital bone; CM2 - cerebellum impacted or herniated into foramen magnum; SM0 - normal; SM1 - central canal dilation or a separate syrinx, which has an internal diameter of less than 2 mm or a pre-syrinx alone; SM2 - syringomyelia (central canal dilation which has an internal diameter of 2 mm or greater, a separate syrinx, or pre-syrinx with central canal dilation). Syringomyelia may be late onset condition so SM0 dogs less than 3 years of age were excluded from statistical calculations for syringomyelia as their clear status could not be assured. Conversely SM1 and SM2 dogs less than 3 years old were included as identifying conformational and genetic indicators of this severe phenotype was a paramount goal of the study.

### Qualitative Observations

**Figure 2:**
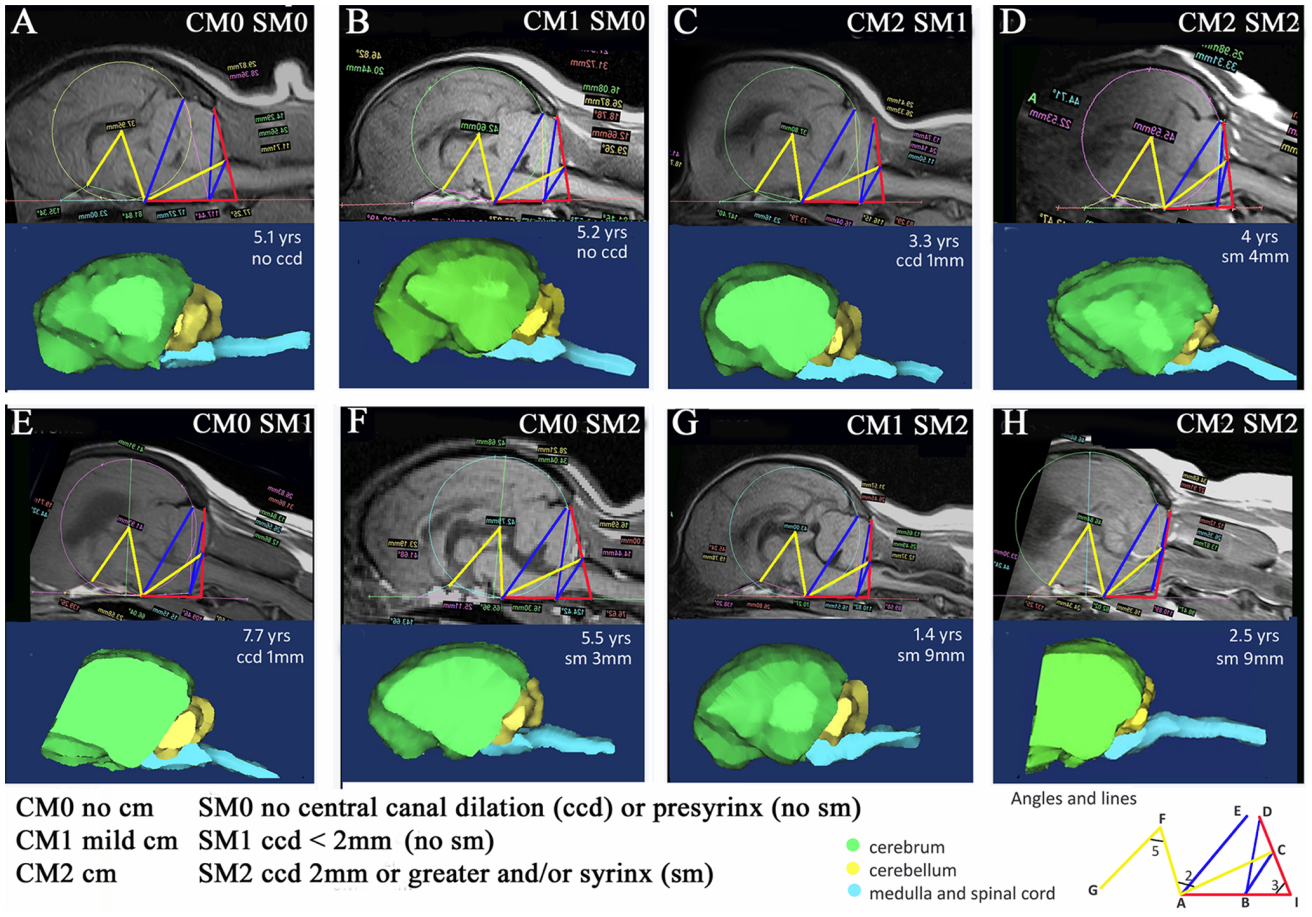
Example Dogs A–H. Top and third rows – T1-weighted midsagittal MRI images with superimposed framework of lines and angles. The yellow lines represent the significant variables in the study: F-diameter and Angle 2 and 5. Angle 3, and Lines AE and BC also found to be significant for CM and syringomyelia and are represented in the red (angle) and blue (lines). From left to right (i.e. Dogs A to D and E to H) there is an increase in CM and syringomyelia affectedness. The second and bottom rows detail the corresponding three dimensional images for each dog constructed from the MRI DICOM. For each dog, the age at time of MRI, maximum transverse width of the central canal dilation or syringomyelia is detailed. CCD – central canal dilatation; Yrs – years; CM – Chiari like malformation; SM – syringomyelia.

Figure 2 and Table 3 illustrate eight representative dogs A-H and their CM and syringomyelia status with increased affectedness from left to right. The MRI images with significant lines and angles and three-dimensional reconstructions of the brain are depicted and dog’s age at time of MRI scan noted. Dogs with CM and syringomyelia tend to have more acute Angles 2 and 5 (colored yellow) and longer lines AE, BC and BD (colored blue). Interestingly this also applied for Dog F which had syringomyelia but was not CM affected. An explanation for this change could be cranial base shortening and increased proximity of the atlas to the occiput. In addition F-diameter was increased suggesting increased height of the rostral cranial fossa. A trend for a decrease in Angle 3 was not a consistent finding and in fact the two dogs with largest Angle 3 (colored red) was Dog E (CM0 SM1) with 1 mm central canal dilation and Dog H (CM2 SM2) with 9 mm syrinx. The three dimensional images illustrate that with increased syringomyelia affectedness the cerebellum is displaced ventral to the occipital lobes giving it a “pushed in” appearance (Dogs D, F, G, and H).

**Table 3:** MRI Measurements of 5 significant variables and CM and syringomyelia status for example dogs A–N.

			MRI measurements (mm)				
Dog (GriffonBruxellois)	CM	SM	F-diameter	AE	angle 2	angle 3	angle 5
A	0	0	38	29.9	81.8	77.3	51
B	1	0	42.6	31.7	76.9	81.2	46.8
C	2	1	37.8	29.4	73.8	83.3	41.5
D	2	2	45.6	33.1	63	83.7	44.7
E	0	1	41.9	31.9	66	91.5	44.3
F	0	2	42.8	34	66	76.6	41.7
G	1	2	43.6	33.2	74	86.2	41.3
H	2	2	46.8	34.7	62	103.9	42.2
**Dog (other breeds)**							
I (Beagle)	0	0	42.72	30.66	80.68	61.37	74.95
J (Aust. Terrier )	0	0	42.26	30.01	80.23	61.67	61.8
K (Aust. Terrier)	1	0	39.54	29.31	72.4	63.78	62.5
L (Affenpinscher)	0	0	40.85	30.95	69.63	88.04	45.75
M (Affenpinscher)	0	0	41.08	31.11	69.42	69.02	45.17
N (Affenpinscher)	1	0	41.32	30.96	60.29	89.08	46.5

Comparison between 8 representative Griffons (A–H) with different degrees of CM and syringomyelia affectedness (0,1,2) and also mesaticephalic and brachycephalic dogs with different degrees of CM affectedness (0,1) but without syringomyelia. Angle 2 becomes more acute with CM and Angle 5 becomes more acute with brachycephaly and this is irrespective of the height of the rostral cranial fossa (F-diameter). Aust. Terrier = Australian Terrier.

**Figure 3:**
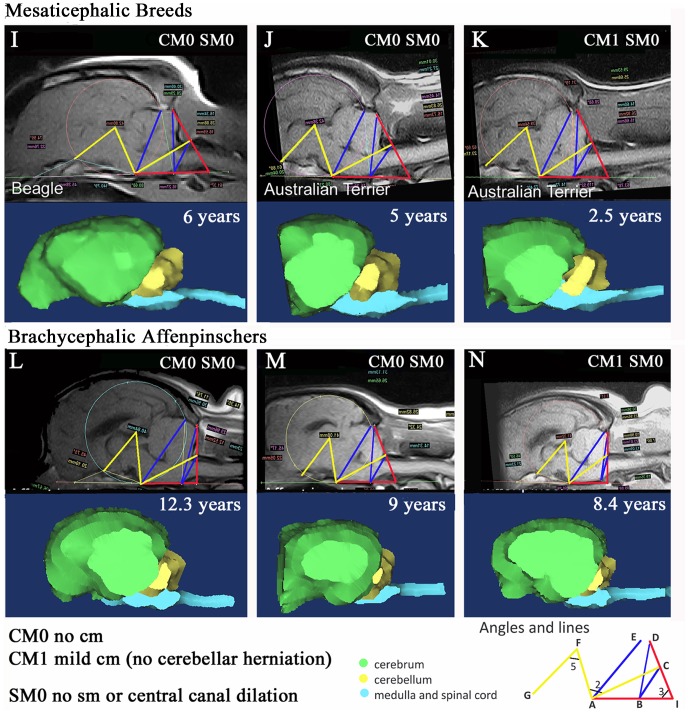
Example Dogs I-N. A comparison of CM between mesaticephalic breeds (Beagle and Australian terrier) and another brachycephalic breed (Affenpinschers). Top and third rows T1-weighted midsagittal MRI image with superimposed framework. The second and bottom rows detail the corresponding three dimensional images for each dog constructed from the MRI DICOM. Yellow: significant angles (Angle 2 and 5). Blue: significant lines (AE, BC, BD). Red: significant Angle 3. The mesaticephalic dogs L,M,N have a smaller angle 3 compared to the brachycephalic dogs I,J,K. Dog K has mild CM and has smaller angles 2 and 5 compared to normal mesaticephalic dogs. Likewise Dog N has mild CM and also has smaller angles 2 and 5 compared to normal brachycephalic dogs. CCD – central canal dilatation; Yrs – years; CM – Chiari like malformation; SM – syringomyelia.

In Figure 3 and Table 3, a comparison is made between mesaticephalic breeds (Beagle and Australian terriers) and a second brachycephalic toy breed (Affenpinscher) which has common ancestors with the Griffon Bruxellois. None of the dogs had syringomyelia thus facilitating a comparison of CM only. Angle 2 becomes more acute with CM which can be appreciated when the normal Beagle (Dog I) and Australian terrier (Dog J) are compared to the Australian terrier with mild CM (Dog K). When the mesaticephalic breeds are compared with the brachycephalic Affenpinchers, Angle 5 becomes smaller and Angle 3 becomes larger. The three dimensional brain images of the CM affected Affenpinschers are similar to the Griffon Bruxellois dogs as the cerebellum becomes “tucked under” and ventral to the occipital lobes.

## Statistical Analysis

### Chiari-like Malformation (CM)

The three groups, CM0 (No CM), CM1 (Mild CM) and CM2 (CM) comprised 31, 32 and 92 dogs respectively. Initial analysis of all possible variables indicated that 8 of the 15 variables were significantly different across the three groups (Table 4). With three groups stepwise Discriminant Analysis produced 2 functions:

**Table 4:** Significant variables identified in the statistical analysis for Chiari-like malformation.

Variable	F	p-value
F-diameter	9.468	<0.001
BC	7.975	0.001
BD	3.828	0.024
AE	5.394	0.006
FG	4.439	0.014
Angle 2	10.552	<0.001
Angle 3	4.299	0.015
Angle 5	4.883	0.009

F is the Analysis of Variance [ANOVA] test statistic. It assesses the ratio of between group variation to within group variation with higher values indicating the likelihood of a group effect.

Function 1 [p<0.001] D1 = − 0.351* F-diameter +0.112* Angle 2+6.968

Function 2 [p = 0.197] D2 = 0.423* F-diameter +0.118* Angle 2–26.357

**Figure 4:**
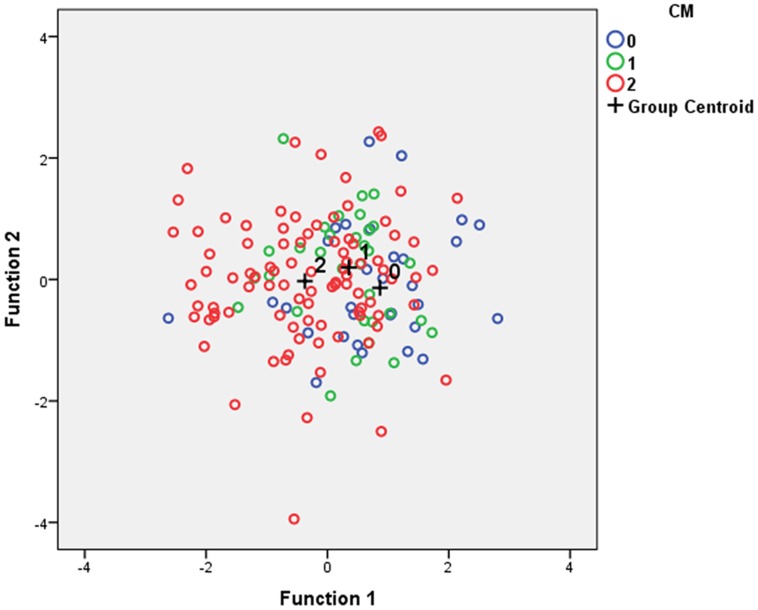
Function1 (horizontal) is plotted against Function 2 [vertical]. The group means (centroid; dark crosses) move more to the left as the CM0 to CM1 to CM2 i.e. CM absent to present.

The stepwise selection confirmed the two most significant variables from the ANOVA results (F-diameter and Angle 2) and the functions themselves are ordered by their level of statistical importance hence Function 1 is the more powerful. Interpreting Function 1 is based on how its value can be increased or decreased hence larger Angle 2 values accompanied by smaller F-diameters will increase this function (larger positive value). Conversely Function 1 will be smaller (more negative) when F-diameter is larger and Angle 2 is smaller. Function 2, whilst statistically non-significant also selected F-diameter and Angle 2 as the important variables and allows for some separation in another dimension, with larger measures of both variables increasing Function 2 values. Figure 4 illustrates this, plotting Function 1 (horizontal) against Function 2 (vertical). The Group means (dark+signs) move more to the left as the CM0 to CM1 to CM2 i.e. CM absent to present. The success of this methodology assessed using cross-validation yielded an overall correct classification result of 52.9% however this success varied across the groups: CM 0 61.3% (19/31); CM 1 40.6% (13/32) and CM2 54.3% (50/92).

### Syringomyelia (SM)

SM0 and SM1 dogs over 3 years were combined to form syringomyelia “SM NO” group ensuring there was no bias for the late onset nature of syringomyelia and SM2 became syringomyelia “SM YES”. Initial analysis of the data from those 135 dogs across all possible variables indicated that 5 of the 15 variables were significantly different (Table 5). Stepwise Discriminant Analysis produces only 1 function when only 2 groups are being compared: Function [p<0.001] D = 0.410 * F-diameter − 0.150 * Angle 5–10.757.

**Table 5:** Significant variables identified in the statistical analysis for syringomyelia.

Variable	F	p-value
F-diameter	20.246	<0.001
BC	4.83	0.03
AE	9.867	0.002
Angle 3	10.769	0.001
Angle 5	13.456	<0.001

F is the Analysis of Variance [ANOVA] test statistic. It assesses the ratio of between group variation to within group variation with higher values indicating the likelihood of a group effect.

**Figure 5:**
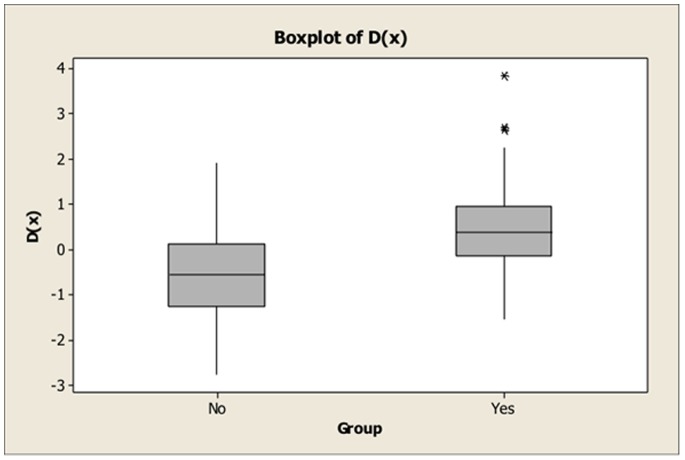
Function values for the SM NO and SM YES groups. The SM YES dogs have slightly higher values of the function (more positive) and the SM NO dogs have slightly lower values. A few SM YES dogs can be seen to be outliers (*) with unusually high function values.

This confirmed the two most significant variables from the ANOVA results (F-diameter and Angle 5). Interpreting the function, as previously, is based on how its value can be increased or decreased hence larger F-diameters accompanied by a smaller Angle 5 will increase the value of the function (more positive) and conversely smaller F-diameters accompanied by a larger Angle 5 will decrease the value of the function (more negative). Figure 5 illustrates these function values for the SM NO and SM YES groups and illustrates that on average the SM YES dogs have slightly higher values of the function (more positive). A few SM YES dogs can be seen to be outliers (*) with unusually high function values.

The success of this methodology was assessed using cross-validation and yielded an overall correct classification result of 69.8%. This success was similar across the groups: SM NO 71.0% (44/62) and SM YES 68.9% (46/67). It was noted that six dogs were dropped from original 135 during the analysis as a result of missing data hence these results are based on 129 dogs.

### Summary of Discriminant Analyses Results

The F-diameter, Angle 2 and Angle 5 variables have been shown to be the significant parameters when aiming to discriminant between the sub-categories of CM and syringomyelia but the power of the discriminatory functions varies considerably, both within a condition and between conditions (Table 6).

**Table 6:** Summary of Discriminant Analyses.

Variable	No CM	CM	No SM	SM
F-diameter	Smaller	Larger	Smaller	Larger
Angle 2	Larger	Smaller	–	–
Angle 5	–	–	Larger	Smaller
‘Success’	61.30%	54.30%	71.00%	68.70%

The variables that yielded the most statistical power of discriminatory functions have been summarized as ‘success’. The magnitude of contribution from each variable is given (Smaller/Larger), with the level of “success” indicating the unbiased rate of dogs correctly classified. CM – Chiari like malformation; SM – Syringomyelia.

### Diagnostic Relevance

**Figure 6:**
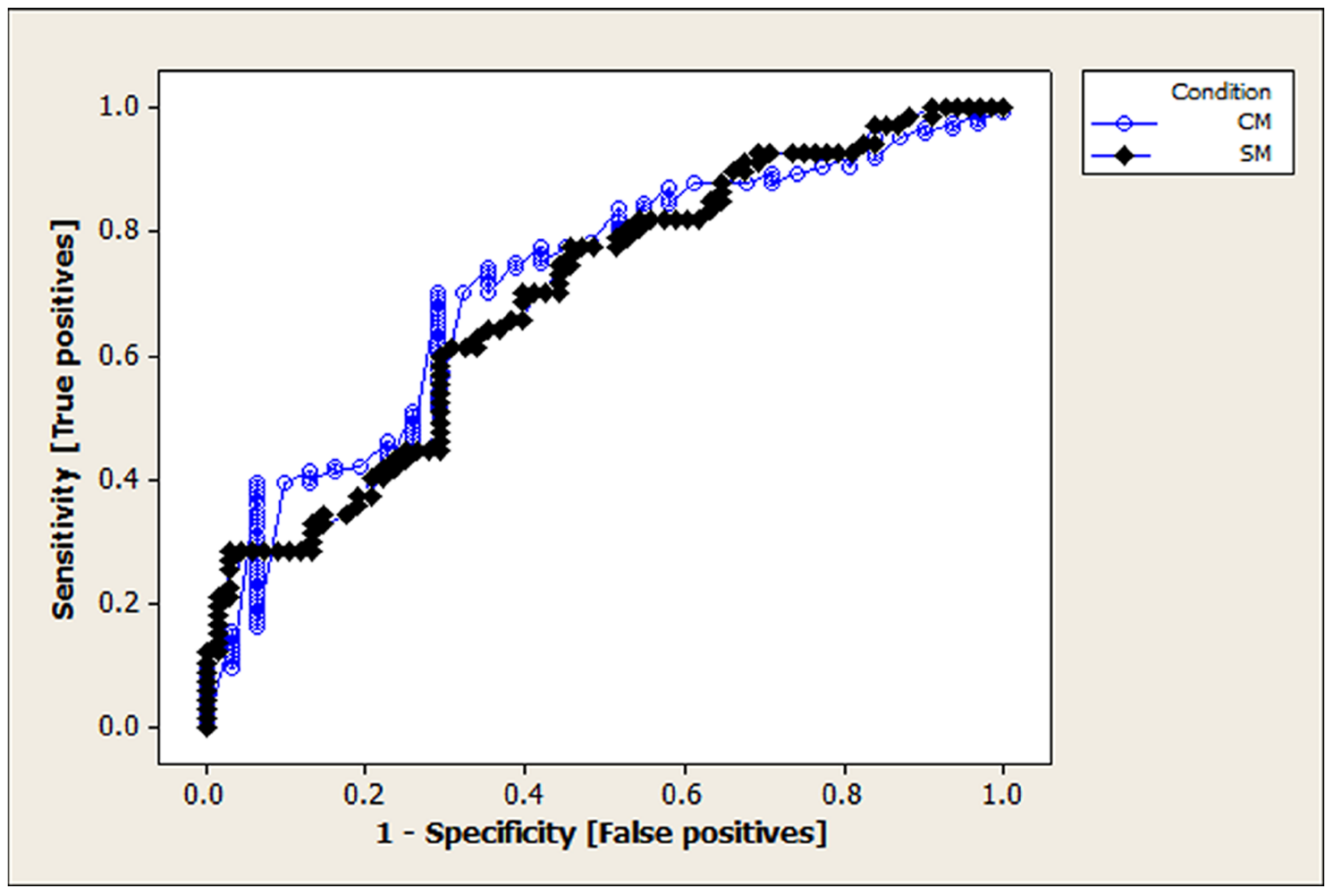
Receiver Operating Characteristic (ROC) curves for CM and syringomyelia based on F-diameter. CM ROC – Area under curve = 0.715 (p<0.001), with estimated optimum cut for F-diameter 41.8 mm; Syringomyelia (SM) ROC – Area under curve = 0.695 (p<0.001), with estimated optimum cut for F-diameter 41.8 mm.

As F-diameter was the most significant variable in each of the previous discriminant analyses, the strength of this variable alone as a diagnostic tool was investigated further. For the 111 dogs where a clear indication of CM NO/CM YES [n = 28, n = 83] and SM NO/SM YES [n = 56, n = 55] was known, very significant statistical differences were found in the average length of F-diameter (p<0.001) for both CM and syringomyelia groupings. From inspection of mean differences a first estimate of an appropriate cut-off for having the condition (or not) was an F-diameter of 42.5 mm. Consequently this estimate was used with 0.1 increases and decreases as a starting point in assessing the sensitivity, specificity, PPV and NPV for both CM (n = 155) and SM (n = 135). ROC analyses produced optimum CM sensitivity of 70%, with specificity 71% and, for SM, a sensitivity of 77%, with specificity of 54%, both occurring at F-diameter of approximately 41.8 mm. Figure 6 details the results. The results indicate high levels of sensitivity, above 70%, are possible for both the diagnosis of CM and syringomyelia. These high sensitivity values are however paired with much lower specificity values around 54% for SM. The PPV results for CM diagnosis was consistently high for F-diameter values in the range but were accompanied by, in general, low NPV. For SM the PPV were in general lower and comparable to the NPV, both being mainly between 60–70%.

## Discussion

### Multifactorial Nature of Chiari-like Malformation

This study supports the multifactorial nature of CM and syringomyelia and that this condition cannot be explained by a simple defect in the development of a single skull bone; it is a more complex disorder involving cranial base shortening, craniocervical junction abnormalities and other, as yet undetermined, factors not investigated in this study. As an example, raised intracranial pressure and/or a mismatch in cerebrospinal fluid (CSF) production and absorption could influence development of syringomyelia but these parameters have yet to be investigated in detail [36], [37]. This study found that decreased Angles 2 and 5, increased diameter of the occipital lobe circle (F-diameter) and, to a lesser extent, decreased Angle 3 and increased AE, BC, BD and FG were the most significant variables. However discriminant analysis could not always predict CM and syringomyelia status suggesting there are other anatomical or environmental factors which affect the development of these disorders. Dogs with CM and syringomyelia were more likely to have increased F-diameter i.e. greater cranial height. This is likely an apparent compensation for cranial base shortening.

### Morphology of Chiari-like Malformation in Brachycephalic Dogs

**Figure 7:**
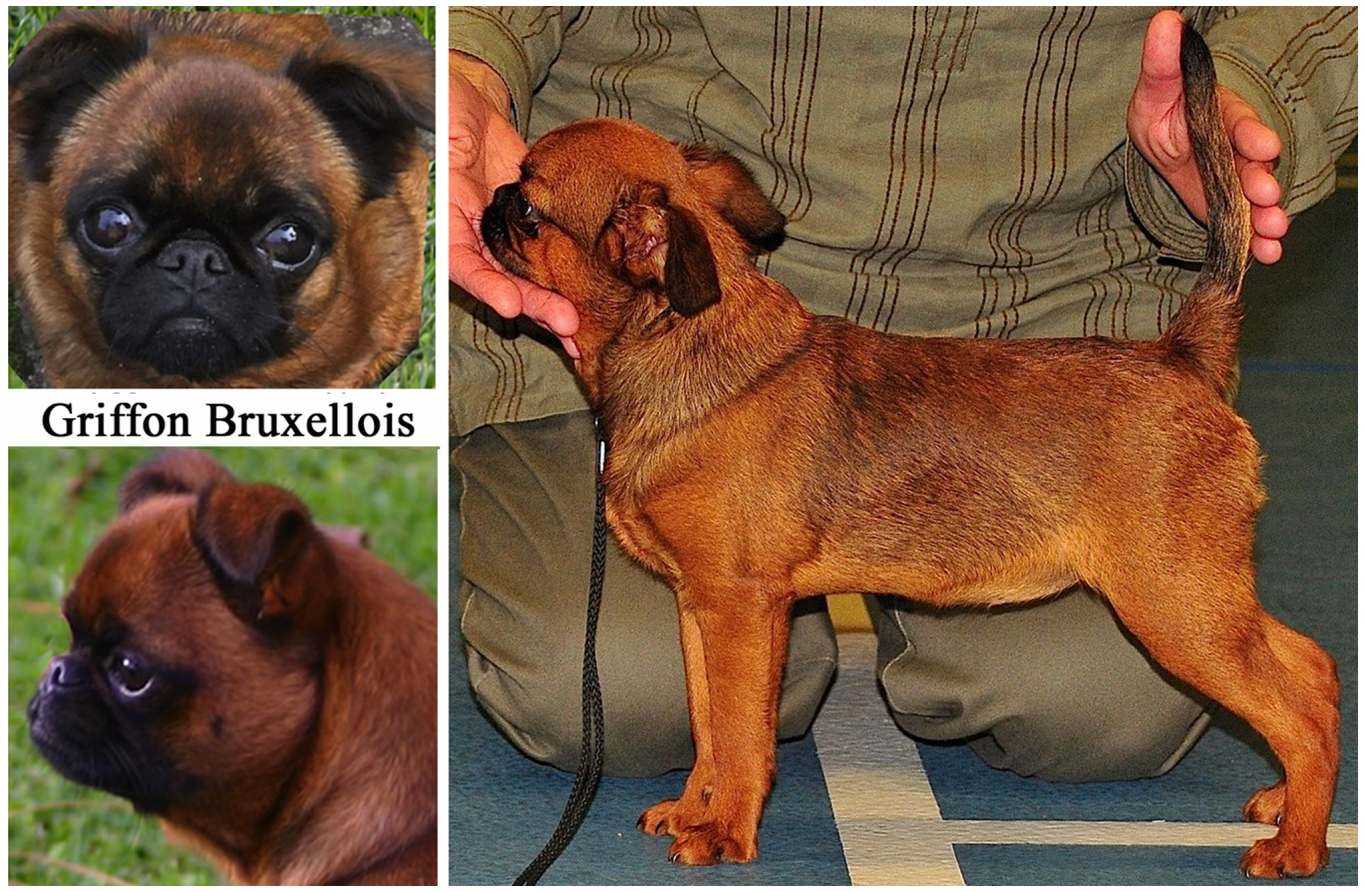
Left : Griffon Bruxellois with ideal head shape and “bombe” (rounded forehead). This dog has syringomyelia but is asymptomatic. To improve success in the show ring breeders may select for greater exaggeration of this characteristic which may increase the risk for symptomatic syringomyelia (picture courtesy of Lee Pieterse). Right: Young Griffon Bruxellois with CM and asymptomatic syringomyelia (picture courtesy of Henny van der Berg).

It is well recognized that brachycephalic dogs have a compensatory increase in cranial height to allow accommodation of the brain in the foreshortened skull [19], [38]. Indeed in their breeding programs, many Griffon Bruxellois breeders select for a “bombe” (rounded forehead) characteristic (Figure 7) (Lee Pieterse personal communication); by doing so it is possible they are selecting for increased risk of CM and syringomyelia.

Line AE was also significantly greater for dogs with CM and syringomyelia. It is possible that this reflects increased height of the rostral part of the caudal cranial fossa (pars rostralis).

**Figure 8:**
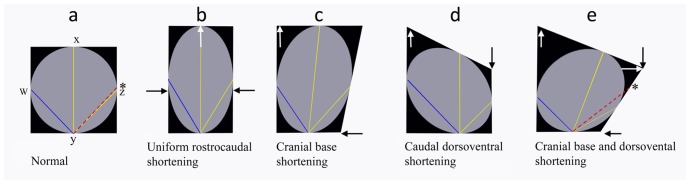
Diagrammatic illustrations to show the hypothetical effects of compensatory changes in skull dimensions and brain shape when there is craniosynostosis or shortening of certain skull bones. These figures were created using Adobe Photoshop (http://www.adobe.com/products/photoshopfamily.html) which enables manipulation of shapes with fixed points facilitating a simple representation of the distortion. a) Normal; the skull is represented by a black box and brain by a grey circle with reference points w, x, y and z. Asterisk – fixed reference point outside brain. b) Uniform rostrocaudal shortening (black arrows). There is a compensatory increase in height of the brain (white arrow). Lines xy, yz (yellow) and wy (blue) increase and angles wyz and xyz decrease c) Cranial base shortening (black arrow); asymmetrical shortening results in an axial tilting of the brain. There is also a compensatory increase in height (white arrow). Line xy and yz increases (yellow), wy shortens (blue) and angle wyx and xyz decrease. d) Caudal dorsoventral shortening (black arrow). There is a compensatory lengthening of the brain and rostral increase in height (white arrow). Line wy increase (blue), xy and yz shorten (yellow), angle xyz decreases e) cranial base and caudal dorsoventral shortening. When the cranial base and the caudal part of the skull are shortened the circular model brain becomes increasing ellipsoid and there is greater axial tilt with an increase in height rostrally (white arrow). The ventorostral part (olfactory bulbs) is displaced ventrally, the dorsocaudal part (occipital lobes) is displaced caudally and the ventrocaudal part (hindbrain) is displaced rostrally. Line yz shortens and angle xyz decreases. However distance to the external reference point (asterisk) increases (red dotted line). This illustration is offered as an explanation for the increase in line AE and BC in this study and also for the change in brain shape with increasing CM affectedness.

It has been shown previously that Cavalier King Charles spaniels with CM have a relatively large volume pars rostralis and relatively small volume pars caudalis [39]. The authors suggested that, in this breed, occipital bone development is insensitive to changes in hindbrain volume and that there is a compensatory bulging of the tentorium cerebelli in a rostral direction [39]. However, an increase in line AE could also occur because of its dependence on the position of the occipital lobes relative to the cerebellum. In dogs with CM the cerebellum appeared to be invaginated under the occipital lobes for example, compare the three dimensional images of Dog A with Dogs D and H in Figure 2. A more acute Angle 2 and longer Line AE with increased rostral cranial fossa height (F-diameter) was also observed in dogs with syringomyelia but without CM e.g. Dog F (Figure 2). Using the term ‘mild’ CM is perhaps misleading if applied to risk of developing syringomyelia. Figure 8 is a schematic representation of the compensatory changes in skull dimensions when there is craniosynostosis or shortening of certain skull bones. The sequence demonstrates how brain parenchyma reorganizes with an increase in cranial height and axial tilt so that the olfactory bulbs shift ventrally and the cerebellum is pushed rostrally. This rotation can mean that measurements between certain fixed points can increase, perhaps explaining the increased length of AE and BC in this study.

### Morphology of Chiari-like Malformation with Syringomyelia

Dogs with CM and syringomyelia were also more likely to have smaller Angles 2, 3 and 5. A more acute Angle 2 could result from a short basicranial axis in the caudal cranial fossa and (even more) premature fusion of the spheno-occipital synchondrosis. Angle 2 was uniquely significant to CM supporting this observation. Angle 2 could also be more acute if the atlas were closer to the occiput either because the supraoccipital bone had lost is convexity i.e. was less rounded or in the event of atlanto-occipital overlapping/proximity. Proximity of the atlas to the occiput would decrease the overall volume of the craniocervical junction; indeed, atlanto-occipital overlapping would not be a prerequisite for this to occur. Reduction in the volume of the craniocervical junction would decrease the size of the subarachnoid space and cisterna magna. This in turn could increase impedance to normal biphasic and pulsatile CSF flow resulting in inhomogeneous flow with abnormal CSF peak velocities predisposing to symptomatic CM and development of syringomyelia. However this hypothesis is not supported by the finding of a significant increase in length of line BC (basion of basioccipital bone to the cranial dorsal laminae of the atlas) which tended to be longer for both CM and syringomyelia groups. In addition Angle 3 tended to be smaller with CM and syringomyelia affectedness - a more acute Angle 3 would occur if the atlas was further away from occiput. However, both BC and Angle 3 was less useful for discriminating dogs with CM and syringomyelia and there are possible alternative explanations for the increase in line BC (Figure 8). A more acute Angle 5 would occur if there was foreshortening of the sphenoid complex suggesting possible insufficiency of this bone or craniosynostosis of the intersphenoidal synchondrosis. A more acute Angle 5 was significant for both syringomyelia and CM and this finding suggests that these disorders in the dog are not just a consequence of a small volume caudal cranial fossa but shortening of the entire cranial base.

### Summary of Conformation Changes with Chiari-like Malformation and Syringomyelia

**Figure 9:**
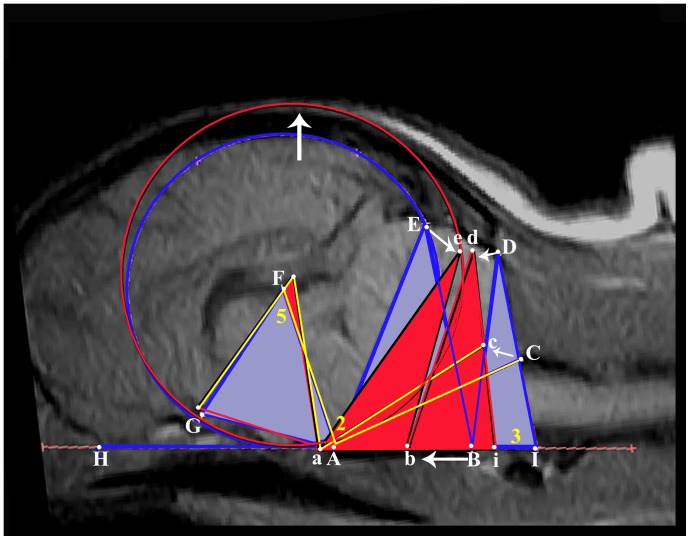
Change in skull and brain conformation in Griffon Bruxellois with CM and syringomyelia. The T1-weighted midsagittal MRI image is from dog A (without CM or syringomyelia) and the framework of lines and angles is indicated in blue and higher case letters with the exception of angles 2 and 5 which are numbered in yellow. The framework of dog D (with CM and syringomyelia) has been superimposed on the image and aligned with baseline HAI and on the F-diameter. The framework of dog D is in red and with lower case letters. Angles 2 and 5 are yellow. It is possible to appreciate how with syringomyelia, the occipital lobe circle and height of cranial fossa increases (red circle). Angles 2 and 5 (yellow) are decreased as a consequence of the cranial base shortening and increased proximity of the atlas to the occiput. In addition the vertexes at E and D (red triangles) are closer together giving an appreciation of the overcrowding and change in shape of the caudal fossa. The white arrows depict the changes between the measurements.

The conformation changes with CM and syringomyelia are summarized in figure 9 which was constructed from the MRI of ‘normal’ Dog A (CM0 SM0) with the significant lines and angles colored blue. The significant lines and angles of Dog D (CM2 SM2) colored red are superimposed on top. One can appreciate the increased height of the rostral cranial cavity (greater diameter of the occipital lobe circle) and decreased in size of angles 2 and 5 (yellow) in compensation for shortening of the entire cranial base and increased proximity of the atlas to the occiput. In a previous study we showed that there is also compensation by lengthening of the parietal bone [3]. Other studies have shown that brachycephalic dogs are more likely to have ventral orientation of the olfactory bulbs [40], [41]. This ventral orientation can be appreciated in the three dimensional images (Figure 2). Our study also suggests that with CM the cerebellum is invaginated under the occipital lobes.

### Value for MRI Screening for Chiari-like Malformation and Syringomyelia

One of the aims of this study was to develop a system of measurements that could be used on MRI obtained by machines of differing field strength and which do not include the entire forebrain. This is necessary because for “low cost” MRI screening a limited but economic imaging is obtained to ascertain CM and syringomyelia status prior to breeding [31]. A typical “low cost” MRI covers an area from the inter-thalamic adhesion to the C4/C5 intervertebral disc space and is often performed on a low field MRI machine. Only sagittal images of the brain are obtained; transverse images are obtained of the spinal cord. Consequently the rostral forebrain is often absent and there are no transverse or dorsal images of the cranium meaning that many measurements are not possible; for example, accurate determination of the skull and parenchyma volume as described in other studies [6]–[9], [30], [39], [42]. We found that using diameter of the occipital lobe circle (F-diameter) allowed an assessment of the height of the cranial cavity even if only the occipital lobes are present. F-diameter alone was a more useful tool for identifying syringomyelia than for CM. A cut-off of 41.8 mm for the F-diameter measure correctly diagnosed around two-thirds of syringomyelia cases with PPV of 61.9% and a NPV of 70.6%. For CM although 85.5% of those known to have the malformation would have been correctly diagnosed when the F-diameter cut-off was 41.8 mm, the NPV was less valuable with approximately half [42.9%] of dogs without CM correctly identified.

Angles proved more useful than point-to-point distant measurements because the latter are affected by head and body size, which is variable even within the same breed. For example we found that the standard error of the caudal skull base length (AB) was almost as large as the actual mean difference hence it was a non-significant. By contrast angles may represent a more accurate representation of the position of one anatomical feature relative to another. However a possible weakness of this study is that the size, weight and body condition of the dogs was unknown and sexual dimorphism was not analyzed which might have affected some parameters.

This study achieved its aim which was to establish a method of qualitative analysis of MRI DICOM images to enable better phenotyping and therefore genotyping. However, it failed to identify a sensitive and specific measurement(s) to improve the current British Veterinary Association and Kennel Club MRI health scheme and better diagnose CM and the risk of syringomyelia at an early age thus precluding the need for dogs to undergo multiple MRI evaluations throughout life. This is most likely because diseases like CM and syringomyelia have a complex etiology and disease expression probably occurs when there is a threshold combination of genetic and environmental factors. Therefore further work is required and we recommend that this study be repeated and/or modified for other breeds with a high prevalence of CM and syringomyelia such as Cavalier King Charles spaniels and Chihuahuas.

## Conclusions

This study supports the view that CM is a multifactorial condition that includes the shortening of the entire basicranium, loss of convexity of the supraoccipital bone, invagination of the cerebellum under the occipital lobes and possibly by increased proximity of the atlas to the occiput. As a compensatory change, there is increased height of the rostral cranial cavity and lengthening of the dorsal cranial vault. Overcrowding in the caudal cranial fossa and the craniocervical junction is a defining feature. The study provides the basis of a quantitative assessment of CM which might identify risk of syringomyelia and suggests that CM should be redefined so that account is taken of the overcrowding of the entire cranial fossa and craniocervical junction with reorganization of the brain.

## Supporting Information

Table S1 **Complete data set of measurements made on 155 dogs in the study.**
(XLSX)

Table S2 **Sensitivity, Specificity, Positive Predictive Value (PPV) and Negative Predictive Value (NPV) based on F-diameter.** The results indicate high levels of sensitivity, above 80%, are possible for both the diagnosis of CM and syringomyelia. These high sensitivity values (PPV) are however paired with much lower specificity values around 55% (NPV). The PPV results for CM diagnosis was consistently high for F-diameter values in the range but were accompanied by, in general, low NPV. For SM the PPV were in general lower and comparable to the NPV, both being mainly between 60–70%.
(XLSX)
